# Imipramine blue sensitively and selectively targets FLT3-ITD positive acute myeloid leukemia cells

**DOI:** 10.1038/s41598-017-04796-1

**Published:** 2017-06-30

**Authors:** Jonathan Metts, Heath L. Bradley, Zhengqi Wang, Neil P. Shah, Reuben Kapur, Jack L. Arbiser, Kevin D. Bunting

**Affiliations:** 1 Department of Pediatrics, Division of Hem/Onc/BMT, Emory University, Atlanta, GA USA; 2 Aflac Cancer and Blood Disorders Center, Children’s Healthcare of Atlanta, Atlanta, GA USA; 3 Department of Medicine, University of California San Francisco, San Francisco, CA USA; 4 Department of Pediatrics, Indiana University, Indianapolis, IA USA; 5 Department of Dermatology, Emory University, Atlanta, GA USA; 6 Atlanta Veterans Administration Medical Center, Decatur, GA 30033 USA

## Abstract

Aberrant cytokine signaling initiated from mutant receptor tyrosine kinases (RTKs) provides critical growth and survival signals in high risk acute myeloid leukemia (AML). Inhibitors to FLT3 have already been tested in clinical trials, however, drug resistance limits clinical efficacy. Mutant receptor tyrosine kinases are mislocalized in the endoplasmic reticulum (ER) of AML and play an important role in the non-canonical activation of signal transducer and activator of transcription 5 (STAT5). Here, we have tested a potent new drug called imipramine blue (IB), which is a chimeric molecule with a dual mechanism of action. At 200–300 nM concentrations, IB is a potent inhibitor of STAT5 through liberation of endogenous phosphatase activity following NADPH oxidase (NOX) inhibition. However, at 75–150 nM concentrations, IB was highly effective at killing mutant FLT3-driven AML cells through a similar mechanism as thapsigargin (TG), involving increased cytosolic calcium. IB also potently inhibited survival of primary human FLT3/ITD^+^ AML cells compared to FLT3/ITD^neg^ cells and spared normal umbilical cord blood cells. Therefore, IB functions through a mechanism involving vulnerability to dysregulated calcium metabolism and the combination of fusing a lipophilic amine to a NOX inhibiting dye shows promise for further pre-clinical development for targeting high risk AML.

## Introduction

Acute myeloid leukemia (AML), the most common leukemia in adults and second most common in pediatrics, is a heterogeneous disease with a high relapse rate. FLT3/ITD mutated AML represents 30% of adult and 10–20% of pediatric AML and despite intensive therapy and allogeneic stem cell transplant the prognosis for these patients remains dismal^1, 2^. FLT3/ITD mutations result in constitutive activation of key survival and growth pathways including STAT5, PI3K, and MAPK. Tyrosine kinase inhibitors (TKIs) that directly target FLT3 (e.g. sorafenib, quizartinib, etc) are currently being studied in clinical trials, however thus far they have produced modest responses, with TKI resistant mutations being a main cause of relapse^3^. Therefore, novel methods to target this high risk disease subset are warranted. Mislocalization of mutant type III receptor tyrosine kinases (RTKs, e.g. FLT3 and c-KIT) occurs in leukemia^4, 5^. In FLT3/ITD^+^ AML, a portion of mislocalized, underglycosylated RTK accumulates on the endoplasmic reticulum (ER) in a highly oxidized microenvironment where the RTK activates signal transducer and activator of transcription 5 (STAT5) through tyrosine phosphorylation, a phenomenon that does not occur with the mature fully glycosylated form, and there are high levels of localized reactive oxygen species (ROS) generated from ER-bound NADPH Oxidase 4 (NOX4)^4, 6^. This non-canonical activation mechanism is leukemia-specific, thus opening the door to an improved therapeutic index for agents that are able to exploit this STAT5 activation mechanism.

ROS are highly reactive molecules containing oxygen. While typically produced by the mitochondria as a byproduct of cellular metabolism, in recent years ROS have been shown to play a key role in many aspects of cell signaling. ROS are elevated in AML and specifically AML with FLT3/ITD mutations^7^. NOX4 has been shown to be a STAT5 target gene, and its upregulation in FLT3/ITD^+^ AML mediates leukemic transformation through inactivation of protein tyrosine phosphatases (PTPs) such as DEP-1 by causing reversible cysteine oxidation at their catalytic site. Furthermore, NOX4 inhibitors have been shown to increase survival in murine models of FLT3/ITD^+^ AML^8^. STAT5 is also directly affected by ROS; in FLT3/ITD^+^ AML cell lines, hydrogen peroxide (H_2_O_2_) co-localizes to the endoplasmic reticulum and is required for STAT5 phosphorylation^9^. These important roles of ROS in FLT3/ITD^+^ AML raise the possibility the ROS may be a therapeutic target for this disease.

Imipramine Blue (IB) is a triphenylmethane dye conjugated to the tricyclic antidepressant imipramine. Many effects of this compound have been discovered, however its most prominent effect is that of NOX inhibition^10^. IB has been shown to be effective in preclinical models of glioblastoma, Burkitt lymphoma, head and neck carcinoma, breast cancer, and chronic myeloid leukemia (CML)^10–14^. Given the prominent role of NOX4 and ROS in FLT3/ITD^+^ AML, we sought to determine if FLT3/ITD^+^ AML might be preferentially sensitive to ROS inhibition. However, we uncovered a new mechanism of action where AML cells are also highly sensitive to cytosolic calcium (Ca^2+^) and vulnerable to Ca^2+^ overload induced mitochondrial cell death^15, 16^.

## Materials and Methods

### Cell culture

All cultured cell lines were grown in media containing 1% penicillin/streptomycin and found to be negative for mycoplasma contamination prior to use. The FLT3/ITD^+^ cell lines MV4–11, MOLM-13, and MOLM-14 were a gift from Doug Graham (Emory University). MV4–11 cells were grown in IMDM supplemented with 10% FBS, and MOLM-13 and MOLM-14 cells were grown in RPMI supplemented with 10% FBS. FLT3/ITD^neg^ cell line OCI-AML3 was purchased from DSMZ and grown in αMEM supplemented with 10% FBS. FLT3/ITD^neg^ cell lines HL-60, HEL, and K562 (blast crisis CML) were gifts from Gang Huang (Cincinnati Children’s Hospital) and William Tse (University of Louisville). HL-60 cells were grown in IMDM with 20% FBS and HEL and K562 cells were grown in RPMI + 10% FBS. MOLM-14 cells with acquired FLT3 point mutations^17^ F691L (gatekeeper mutation) and D835Y (tyrosine kinase domain mutation) were grown in RPMI + 10% FBS. 32D clone 3 cells transduced with wild-type c-KIT and c-KIT D814V mutation were grown in IMDM + 10% FBS supplemented with 5 ng/mL murine IL-3 (Gemini) and 32D KIT D814V cells were starved of IL-3 for at least 24 hours prior to experimentation.

### Human umbilical cord blood and primary patient samples

Umbilical cord blood units were obtained with IRB approval from the Carolinas Cord Blood Bank (Duke University). All methods were carried out in accordance with relevant guidelines and regulation. Informed consent was obtained for all subjects donating cord blood units to the bank. Mononuclear cells were extracted using Ficoll then counted via trypan blue to assess viability. CD34^+^ cells were then extracted utilizing the human UltraPure CD34 MicroBead Kit (Miltenyi, 130-100-453). Cells were expanded as described^18^ in Stemspan H3000 serum free media (StemCell Technologies, #09800) supplemented with 100 ng/mL of human thrombopoietin, IL-6, FLT3 ligand, stem cell factor (Gemini) and 1 µM StemRegenin-1 (SR-1, Cayman Chemicals, #10625).

De-identified patient samples were obtained from the Children’s Oncology Group (COG) through the AML Biology Committee (AAML15B9-Q). Bone marrow samples containing 70% blasts or greater were from patients under 21 years of age at diagnosis prior to receiving any therapy. Samples were thawed and expanded in cytokines, SR-1, and UM-729 (StemCell, #72332) as described^19^. Samples were considered satisfactory quality for analysis if >50% viability was maintained after 5 days in culture.

### Treatment reagents

Mitochondrial complex I inhibitors rotenone and metformin (#R8875 and # D150959), NOX inhibitor diphenyleneiodonium (DPI, #D2926), SERCA inhibitor thapsigargin (T9033), and STAT5 inhibitor pimozide (P1793) were purchased from Sigma. The lipophilic amine imipramine and the triphenylmethane dyes gentian violet and imipramine blue were obtained from Sigma. Imipramine blue was prepared at PCI Synthesis, Newburyport, MA. PCI is a cGMP facility which has prepared imipramine blue, and confirms the identity of imipramine blue by mass spectroscopy and NMR spectroscopy.

### Western blotting

Cells were lysed in RIPA buffer with protease and phosphatase inhibitors (Roche, #04693124001 and #04906845001) for 30 minutes on ice followed by centrifugation for 10 minutes at 10,000 × g. Protein concentrations were determined using the Bio-Rad protein assay (Bio-Rad, # 500–0006) and proteins were separated on an SDS-polyacrylamide gel followed by transfer to either a nitrocellulose (Fisher, #1215471) or PVDF (Immobilon, #IPVH00010) membrane. After blocking in 5% BSA for 1 hour, membranes were incubated in the appropriate antibody overnight and detection was by chemiluminescence (Thermo Scientific, #34080) or by the Odyssey Clx imaging system (LI-COR Biosciences). Image Studio v4.0 software was used for densitometry quantification.

### Assays of mitochondrial integrity

For ROS detection, cells were washed twice with PBS then incubated for 30 minutes in 1 µM H2-DCF-DA (Invitrogen, D-399) in PBS. After 2 more washes in PBS, hydrogen peroxide levels were detected by flow cytometry. For mitochondrial membrane potential, after 2 washes with PBS, cells were incubated with 5 µM JC-1 (Thermo-Fisher, T3168) in PBS for 20 minutes, then washed 3 times with PBS prior to analysis by flow cytometry^20^.

### Cytoplasmic Calcium Assay

After 2 washes with serum-free RPMI medium, cells were incubated with serum-free RPMI with 5 µM Indo-1 AM (Thermo-Fisher, I1203) for 30 minutes. Cells were then washed 3 times with PBS. Flow cytometry was performed by ratiometric analysis of cytoplasmic calcium-bound vs unbound Indo-1 vs time. Analysis was performed for 30 seconds on untreated cells prior to the addition of treatment agents.

### Cell Imaging

The fluorescent properties of IB were utilized for localization analyses. IB is excited at 568 and emits fluorescence at >650. ER-tracker Green was purchased from Thermo-Fisher (E34250) and Golgi-ID and Lyso-ID were purchased from Enzo (ENZ-51028-K100 and ENZ-51034-0100). These dyes were chosen because they have no spectral overlap with IB. Hoechst stain provided in the Enzo kits was used for nuclear counterstain. Staining was performed according to manufacturer’s protocols. Cells were concurrently loaded with 150 nM IB. High resolution live-cell imaging was performed on the DeltaVision OMX (GE Healthcare). Post-acquisition data analysis was done using Fiji software (Fiji.sc).

### Cell survival assays

For apoptosis assay, after 2 washes in PBS, cells were resuspended in Annexin Buffer with Annexin V-PE (BD Biosciences, 556421) and DAPI, according to the manufacturers’ instructions. Cells were analyzed by flow cytometry. For cytotoxicity assay, after 48 hours of drug treatments, cells were analyzed by trypan blue exclusion assay as previously described^21^.

### Statistical Analysis

All data were derived as a result of three or more independent experiments, unless stated otherwise. Student’s two tailed t-test was used to calculate p-values and values less than 0.05 were considered to be significant.

## Results

### Inhibition of ROS potently inhibits STAT5 phosphorylation but induces cell death at lower concentrations

**Figure 1:**
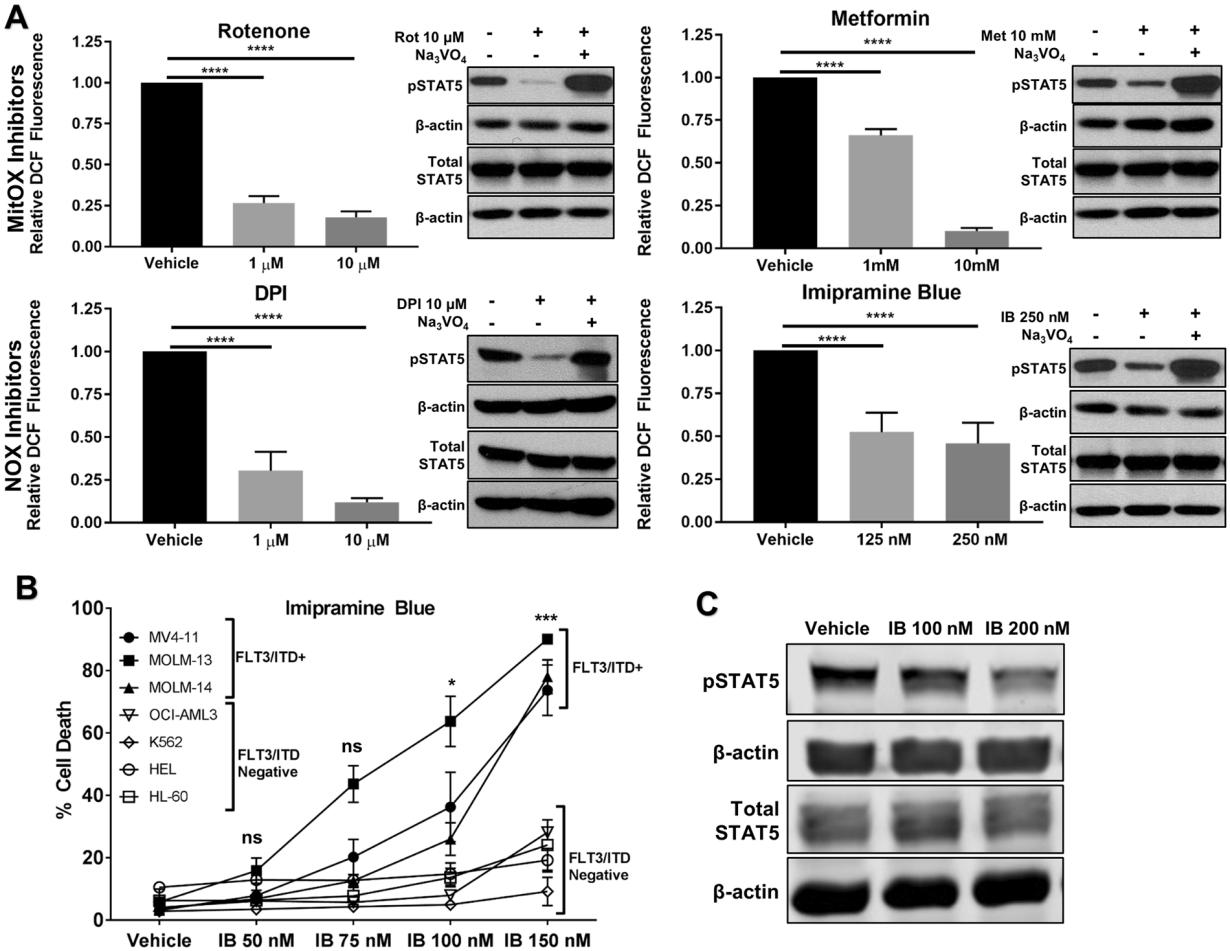
Imipramine blue selectively kills FLT3/ITD^+^ AML cell lines at lower concentrations than required to suppress STAT5 phosphorylation. (**A**) MV4–11 cells were treated with mitochondrial complex I inhibitors (rotenone and metformin) or NOX inhibitors (DPI and imipramine blue) for 4 hours prior to 30 minute H2-DCF-DA staining and flow cytometry. For Western blots, MV4–11 cells were treated for 4 hours with or without 1 mM PTP inhibitor sodium orthovanadate (Na_3_VO_4_). MitOX inhibitors decreased pSTAT5, and this effect was reversed in the presence of Na_3_VO_4_, indicating PTP liberation as the cause of decreased pSTAT5. (**B**) Trypan blue exclusion assay after 48 hours drug exposure. FLT3/ITD^+^ cell lines were selectively killed at nM doses of IB, specifically 100–150 nM. (**C**) Doses of 200 nM IB were required to see significant inhibition of pSTAT5. The original uncropped Western blot files are included as Supplemental material. (*p < 0.05, ***p < 0.001, ****p < 0.0001, ns: p > 0.05).

To determine the effects of ROS inhibition on STAT5 phosphorylation, we targeted the 2 main cellular ROS producers: mitochondria and NADPH oxidase. Utilizing mitochondria complex I (MitOX) inhibitors (rotenone, metformin) and NADPH Oxidase (NOX) inhibitors (DPI, imipramine blue) for 4 hours, cellular levels of ROS as measured by hydrogen peroxide content in MV4–11 cells were effectively decreased. Western blot after 4 hour treatment demonstrated corresponding inhibition of pSTAT5 (Fig. 1A). Effective disruption of downstream STAT5 signaling was also observed using qRT-PCR for STAT5 target genes (Fig. S1). Co-treatment with 1 mM sodium orthovanadate (Na_3_VO_4_), a protein tyrosine phosphatase (PTP) inhibitor restored pSTAT5 levels as measured by Western blot, implicating the liberation of PTPs by ROS inhibition as an effective method of pSTAT5 inhibition. (Fig.1A).

### IB induces apoptosis that is selective for FLT3/ITD^+^ cell lines, alone and in combination with pimozide

**Figure 2:**
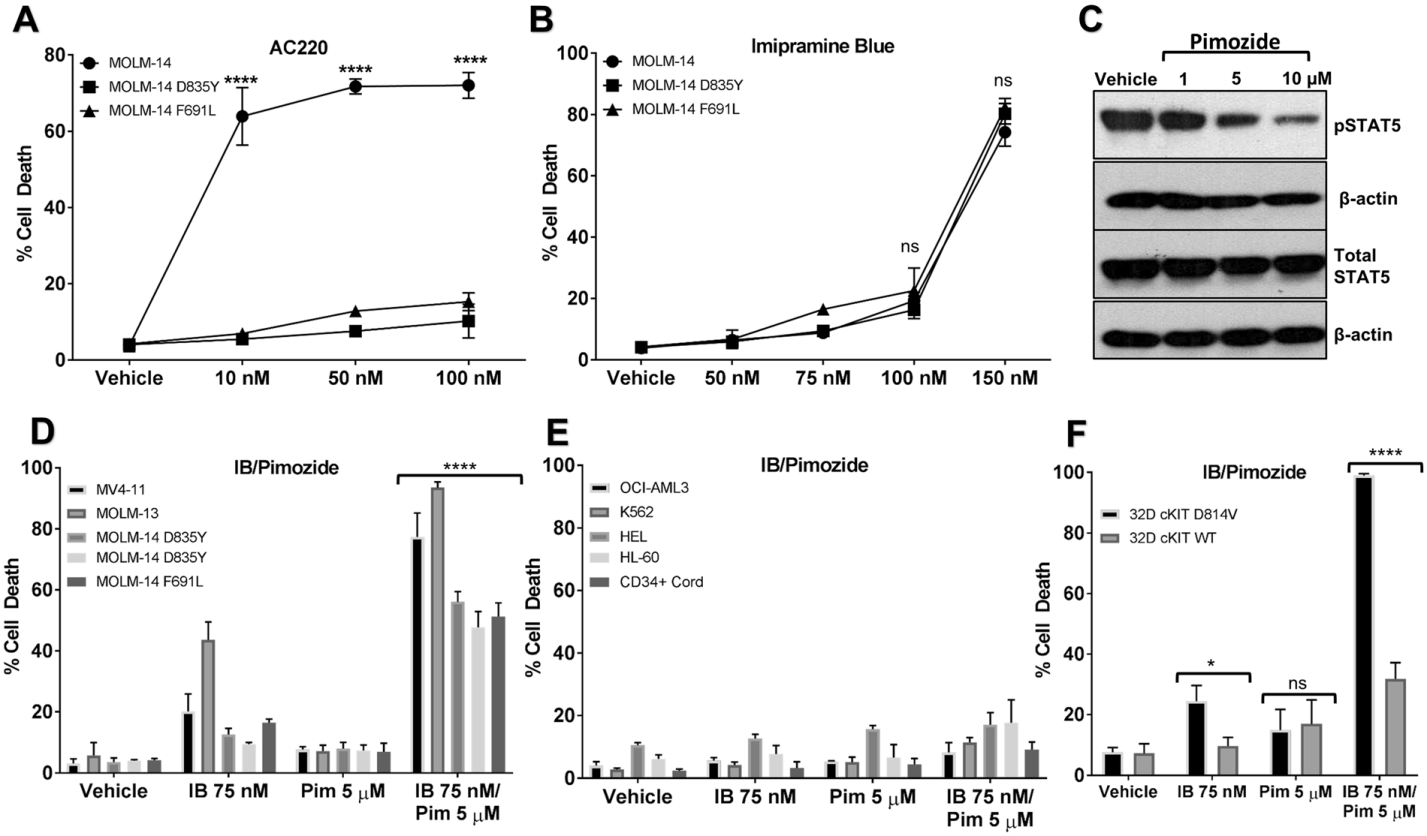
Imipramine blue targets AC220-resistant FLT3/ITD mutants and synergizes with pimozide to selectively kill FLT3/ITD and KIT/D814V expressing cells. (**A** and **B**) By trypan blue exclusion assay at 48 hours of drug treatment, MOLM-14 cells with acquired point mutations in the tyrosine kinase domain (D835Y) and gatekeeper domain (F691L) were resistant to AC-220, but demonstrated no resistance to IB as compared to the parental MOLM-14 line. (**C**) Pimozide inhibited pSTAT5 at μM doses in MV4–11 cells. (**D** and **E**) Trypan blue exclusion assay at 48 hours showed that in combination, sub-optimal doses of IB and pimozide were highly synergistic and selective in FLT3/ITD^+^ cell lines, including those with FLT3 point mutations, with little effect on FLT3/ITD^neg^ cell lines or on CD34^+^ cord blood cells. (**F**) 32D cells transduced with c-KIT D814V mutation were also sensitive to IB/Pimozide. The original uncropped Western blot files are included as Supplemental material. (*p < 0.05, ****p < 0.0001, ns: p > 0.05).

Next we determined the effects of ROS inhibitors on AML cell death. With 48-hour drug treatment, the MitOX inhibitors and the NOX inhibitor DPI did not show selectivity for FLT3/ITD^+^ cell lines (Fig. S2A–C). However, IB at 48 hours demonstrated potent and selective cell death for FLT3/ITD^+^ cell lines tested, with relatively little effect on FLT3/ITD^neg^ lines (Fig. 1B) Surprisingly, the IC_50_ values for the FLT3/ITD^+^ cell lines were between 100–150 nM, below the dose of IB where pSTAT5 inhibition was achieved (Fig. 1C). Therefore, we hypothesized that the mechanism of cell death was independent of direct FLT3/ITD/pSTAT5 inhibition and AML which becomes resistant to TKIs may remain sensitive to IB. We tested MOLM-14 cells which were transduced with acquired FLT3 point mutations that occur clinically and confer TKI resistance. Cell lines with these point mutations demonstrated resistance to the highly specific FLT3 inhibitor AC-220, but conferred no resistance to IB. (Fig. 2A,B).

To optimize selectivity and FLT3/ITD^+^ cytotoxicity, we also combined IB with the STAT5 inhibitor pimozide, which inhibits pSTAT5 at doses ≥5 µM in the MV4–11 cell line (Fig. 2C)^22, 23^. Doses that were suboptimal for killing FLT3/ITD^+^ cell lines at 48 hours were surprisingly selective and synergistic for FLT3/ITD^+^ cell lines, sparing FLT3/ITD negative cell lines and CD34^+^ cord blood cells (Fig. 2D,E). The combination was also selective and synergistic for 32D cells transduced with c-KIT D814V mutant when compared to 32D cells with wild-type c-KIT (Fig. 2F).

**Figure 3:**
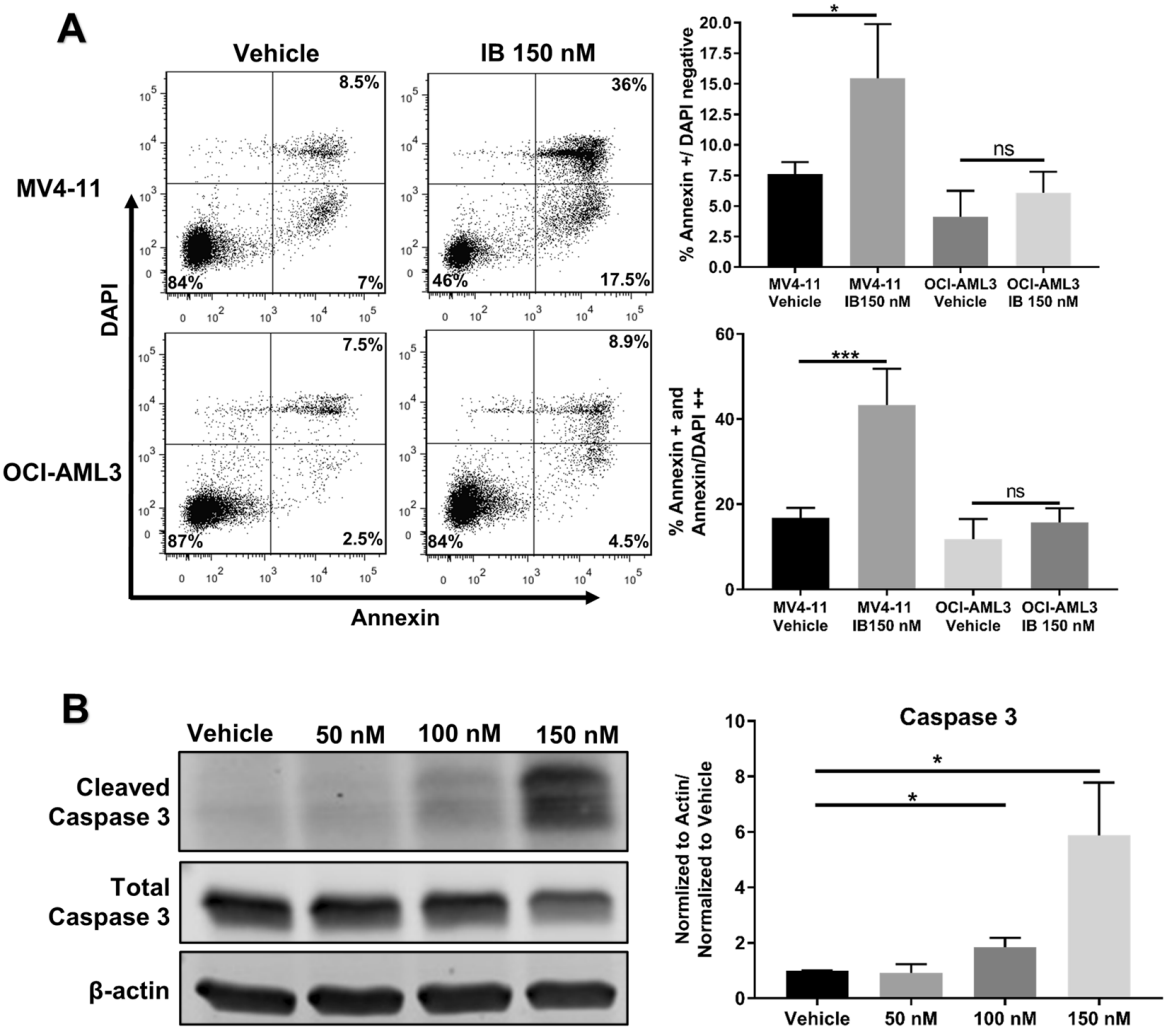
Imipramine blue targets FLT3/ITD^+^ AML cells by inducing caspase 3 cleavage and apoptosis. (**A**) After 18 hours of drug treatment, Annexin V/DAPI assay demonstrated a population of Annexin V^+^/DAPI^neg^ and a population of Annexin V^+^/DAPI^+^ cells that was statistically significant for MV4–11 cells but not for OCI-AML3, indicating the IB was selectively inducing cell death through apoptosis (**B**) Western blot after 24 hours of drug treatment in MV4–11 cells demonstrated significant Caspase 3 cleavage at doses of 100 and 150 nM IB, indicating induction of apoptosis as a mechanism of IB-mediated cell death. The original uncropped Western blot files are included as Supplemental material. (*p < 0.05, ***p < 0.001, ns: p > 0.05).

We next determined the role of apoptosis in IB cytotoxicity. Annexin V/DAPI staining after 18 hours of drug treatment caused significant increases in the Annexin single positive and Annexin/DAPI double positive populations in MV4–11 cells with little effect on OCI-AML3 cells, indicating a selective cell death through apoptosis in the FLT3/ITD^+^ cell line (Fig. 3A). This was confirmed by significantly increased cleavage of Caspase 3 in MV4–11 cells treated for 24 hours (Fig. 3B).

### IB co-localizes to lysosomes and causes cytosolic Ca^2+^ release

**Figure 4:**
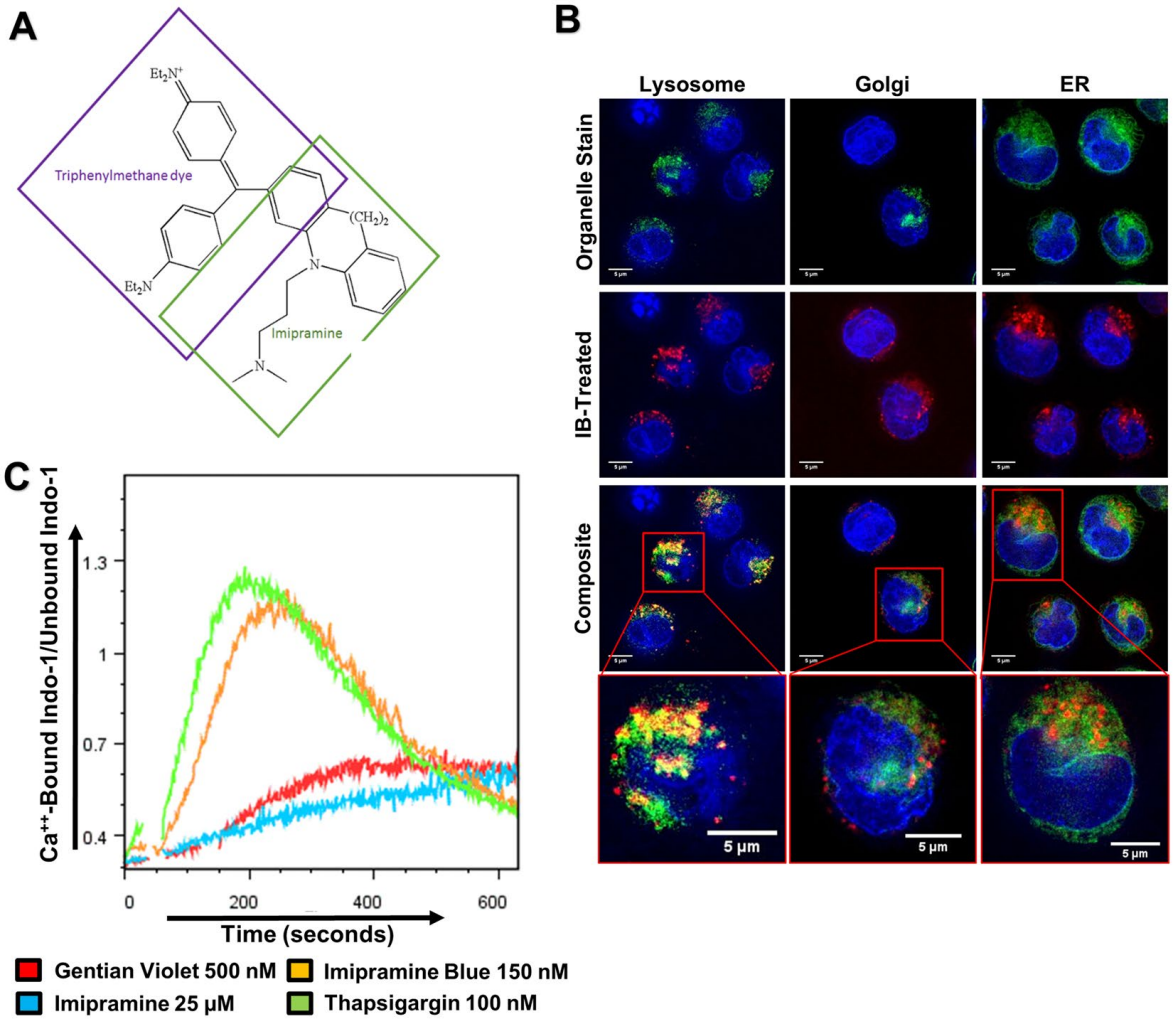
Imipramine blue is trapped in the lysosomes and increases cytosolic calcium concentration comparable to thapsigargin. (**A**) Molecular structure of IB, demonstrating the conjugation of Imipramine to a triphenylmethane dye. (**B**) (Representative result) Live cell imaging of organelle trackers and IB demonstrated a co-localization of 150 nM IB with lysosomal tracker, which was not seen with ER and Golgi tracker. (**C**) (Representative result) IB and the SERCA inhibitor TG caused a surge in cytoplasmic Ca^2+^ while Imipramine and Gentian Violet showed minimal effect on Ca^2+^ release.

Given the properties of IB as a lipophilic amine moiety conjugated to a triphenylmethane dye, we next hypothesized that cellular organelles may have disproportionately higher uptake of IB, leading to organelle stress (Fig. 4A). To determine if a specific organelle is targeted by IB, we utilized the fluorescent properties for cellular imaging. Organelle tracking dyes were used to determine cellular localization of organelles with substantial Ca^2+^ stores: ER, Golgi complex, and lysosomes. We found IB to localize outside of the nucleus in a punctate pattern. On dual ER/IB localization, we noted that IB localized to the same region of cells, but did not overlap with the ER or the Golgi (Fig. 4B). However there was a high degree of co-localization observed with lysosomes. This is consistent with the previously described phenomenon of “lysosomal trapping” of lipophilic amines due to the acidic environment of the lysosome^24^.

**Figure 5:**
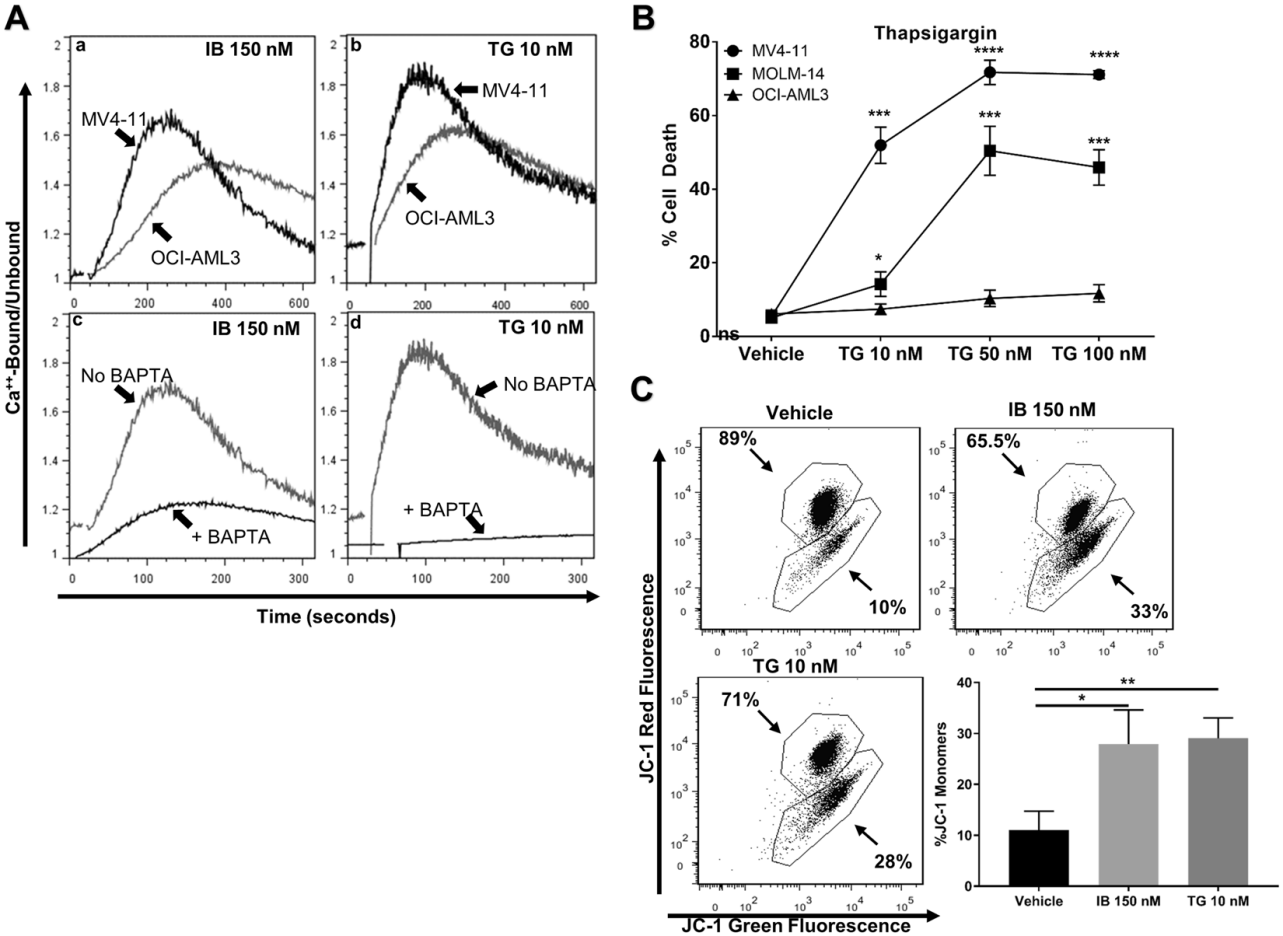
Low dose imipramine blue-mediated calcium induced cell death and both imipramine blue and thapsigargin are potent inducers of mitochondrial outer membrane permeabilization. (**A**) (Representative result of n = 3) Indo-1 cytoplasmic Ca^2+^ assay. IB and TG caused Ca^2+^ release in OCI-AML3 cells to a lesser extent (a,b). In MV4–11 cells, IB and TG demonstrated an influx of cytoplasmic Ca^2+^ that was reversed by the addition of 5 μM BAPTA (c, d) (**B**) Trypan blue exclusion assay after 48 hours of drug treatment showed that TG has a similar selective cytotoxicity profile as IB for FLT3/ITD^+^ cell lines, implicating Ca^2+^ release in the mechanism of cell death. (**C**) JC-1 assay demonstrated a significant increase in JC-1 monomers after 18 hours of drug treatment with IB or TG in MV4–11 cells. (*p < 0.05, **p < 0.01, ***p < 0.001, ****p < 0.0001, ns: p > 0.05).

We next evaluated the effects of IB on cytosolic calcium release utilizing the Indo-1 assay. IB induced Ca^2+^ release in MV4–11 cells as did the positive control thapsigargin (TG), a SERCA inhibitor which blocks Ca^2+^ reuptake by the ER and Golgi complex (Fig. 4C). Gentian violet, a triphenylmethane dye without a conjugated amine did not induce Ca^2+^ release, nor did the IB pro-drug imipramine, demonstrating that this is a unique characteristic of IB (Fig. 4C). IB also induced calcium release in OCI-AML3 cells, indicating this is not a FLT3/ITD-specific phenomenon (Fig. 5A). However, the peak Indo-1 bound/unbound ratio for OCI-AML3 cells was not as remarkable as for MV4–11 cells, indicating that the Ca^2+^ release may be more dramatic in FLT3/ITD^+^ cells. Ca^2+^ release and overload of the mitochondria is known to induce a loss of mitochondrial membrane potential (ΔΨm) and apoptosis. We then treated cell lines for 48 hours with TG and saw a similar sensitive and selective cytotoxicity for FLT3/ITD^+^ cell lines, confirming the role of cytoplasmic Ca^2+^ (Fig. 5B). We therefore examined the effects of IB and TG on ΔΨm. Both IB and TG caused an increase in JC-1 monomers, indicating that Ca^2+^ release is causing a loss of mitochondrial membrane potential, resulting in mitochondria-induced (intrinsic) apoptosis (Fig. 5C).

### FLT3/ITD^+^ patient samples are more sensitive to IB than FLT3/ITD^neg^ samples

**Figure 6:**
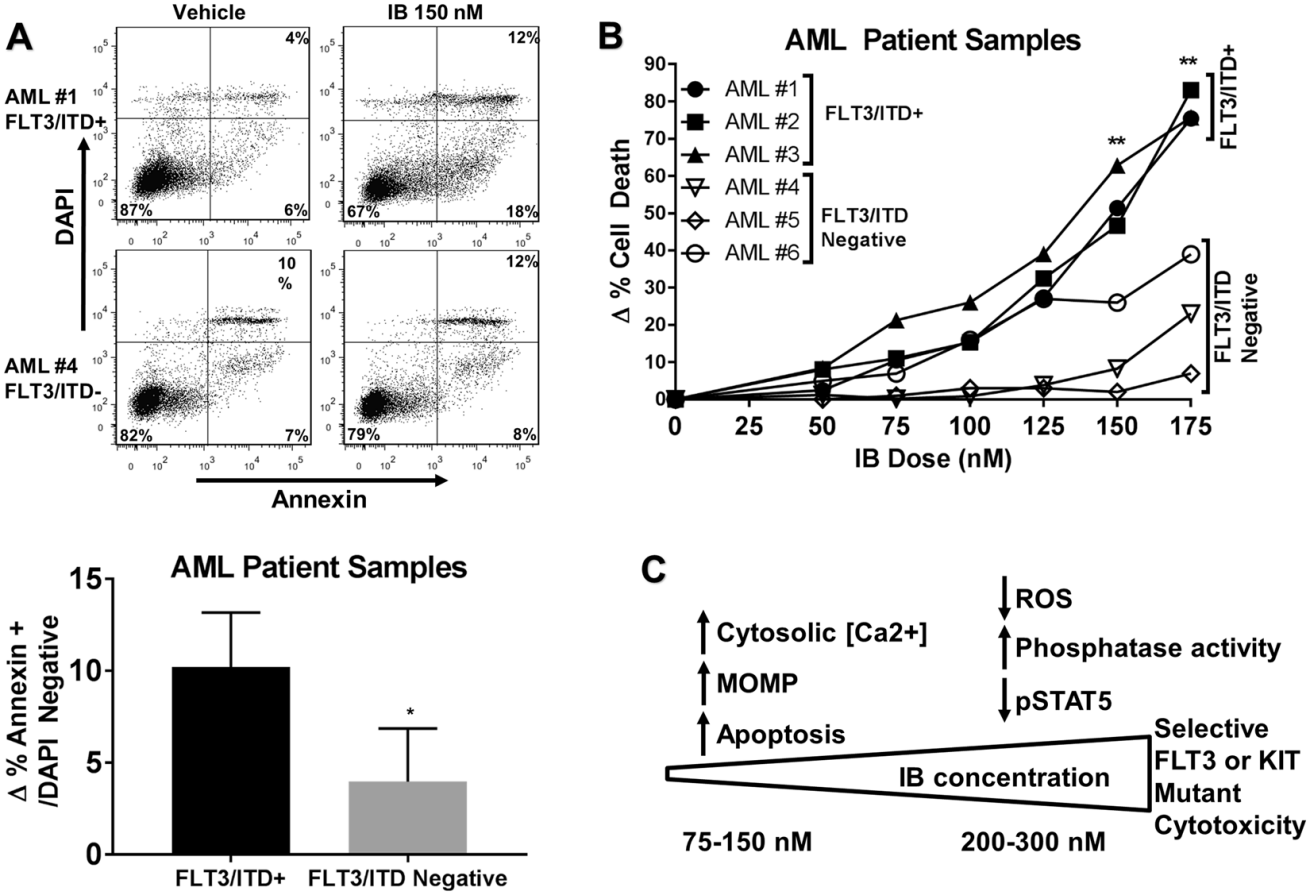
Imipramine blue is an effective inducer of apoptosis and selective killing of primary human FLT3/ITD^+^ AML cells compared with FLT3/ITD^neg^ AML cells. (**A**) After 18 hours treatment with 150 nM IB, FLT3/ITD^+^ samples demonstrate apoptosis by Annexin V single positive staining, which was not seen in FLT3/ITD^neg^ samples. (**B**) 48 hour trypan blue exclusion assay showed sensitivity to IB for FLT3/ITD^+^ at similar doses to cell lines. (**C**) Two-fold dose-dependent mechanism for IB-induced FLT3/ITD^+^ AML cell death. (*p < 0.05, **p < 0.01, ns: p > 0.05).

Utilizing primary pediatric AML samples obtained at the time of diagnosis in *de novo* AML patients, we next determined the effects of IB treatment. Annexin V flow assay demonstrated a larger population of Annexin V^+^/DAPI^neg^ cells at 18 hours of 150 nM IB treatment that trended toward statistical significance (p = 0.06). FLT3/ITD^neg^ samples remained relatively unaffected (Fig. 6A). At 48 hours post-treatment, trypan blue assay showed that FLT3/ITD^+^ samples were sensitive to IB in similar doses to FLT3/ITD^+^ cell lines while FLT3/ITD^neg^ samples were not, demonstrating that IB effectively targets more heterogeneous populations of primary FLT3/ITD^+^ AML cells (Fig. 6B).

## Discussion

AML with the FLT3/ITD mutation portends a poor prognosis. The mutation alone is insufficient to generate an acute leukemic transformation but induces myelo- and lymphoproliferative disease and FLT3/ITD has been validated as a therapeutic target^17, 25, 26^. Both multikinase (e.g. sorafenib) and more selective class III receptor tyrosine kinase inhibitors (TKIs) (e.g. quizartinib) have now reached early-phase clinical trials, however durable remissions with TKI monotherapy have been limited. Multiple mechanisms are responsible for acquired TKI resistance, with the most prominent being the acquisition or expansion of AML clones with the ITD as well as TKD point mutations^27^. Current translational and clinical efforts targeting FLT3/ITD expressing cells include the development of more robust TKIs (e.g. crenolanib) and utilization of TKIs in combination with chemotherapy backbones or following hematopoietic stem cell transplantation as a maintenance therapy^28^. Given the numerous proliferative and anti-apoptotic downstream pathways activated by FLT3/ITD (STAT5, PI3K, MAPK), targeting of downstream lesions is an attractive alternative to direct inhibition of FLT3/ITD.

The data presented here reveals that the NOX inhibitor IB induces a potent and selective cell death to FLT3/ITD^+^ AML through dual mechanisms of ROS/STAT5 inhibition as well as lysosomal Ca^2+^ release. Through the decreased production of NOX-derived ROS, IB potently inhibits the tyrosine phosphorylation and activation of STAT5 at doses of approximately 200–300 nM. Surprisingly, IB was highly selective against FLT3/ITD^+^ AML at 75–150 nM doses, which induced cytosolic Ca^2+^ release from lysosomes, resulting in mitochondrial outer membrane permeabilization, caspase activation, and intrinsic apoptosis^29^. The therapeutic strategy of cytosolic Ca^2+^ release was confirmed through SERCA inhibition with TG, which also resulted in potent and selective cell death of FLT3/ITD^+^ cells. Ca^2+^ release may occur via the inhibition of calcium re-uptake channels (e.g. SERCA inhibitors) or through the disruption of Ca^2+^-storing organelles (i.e. the endoplasmic reticulum; ER, Golgi apparatus, and lysosomes)^30, 31^. We also discovered a novel synergistic combination of IB with the STAT5 inhibitor pimozide which effectively targets FLT3/ITD^+^ AML while sparing cell lines without the mutation and sparing CD34^+^ cord blood cells. Importantly for IB monotherapy and the IB/pimozide combination, no drug resistance was observed for FLT3/ITD^+^ cell lines with acquired TKD mutations, indicating that IB and IB/pimozide may be able to overcome this substantial hurdle to FLT3/ITD^+^ AML treatment. IB also effectively induced cytotoxicity in FLT3/ITD^+^ primary patient samples, indicating this therapeutic strategy has potential in more heterogeneous leukemic cell populations. The dual-effects of IB cytotoxicity and the synergistic combination of IB/pimozide are attractive therapeutic strategies because of their selectivity for FLT3/ITD mutations, and there is likely less potential for development of drug resistance.

STAT5, a crucial transcription factor for hematopoiesis, is activated through tyrosine phosphorylation. Because of its broad roles in hematopoiesis and hematopoietic stem cells (HSCs), our lab and others have shown that deletion of STAT5 causes significant immune suppression, multilineage cytopenias, and stem cell dysfunction, thus far limiting the feasibility of STAT5 as a druggable target. STAT5 is classically activated through the canonical JAK-STAT pathway. However, STAT5 activation can be uncoupled from canonical JAK2-STAT5 activation^32^. Its downstream targets include several anti-apoptotic and pro-survival genes (i.e. c-Myc, Pim-1, etc) that are known to be associated with oncogenic transformation^33^. STAT5 is downstream of many oncogenic tyrosine kinases including FLT3/ITD, BCR-ABL, JAK2^V617F^, KIT^D816V^ and others, making it an attractive target for anti-leukemic therapy^34^. Unfortunately, STAT5 inhibition as a therapeutic target has been difficult to achieve and there is lack of preclinical and clinical agents with the capability to directly target STAT5, although there are now some new compounds with advantages and some limitations^35–37^. Typically STAT5 inhibition occurs through the inhibition of upstream tyrosine kinase pathways and inhibitors to FLT3, JAKs, and BCR-ABL may lead to drug resistance.

ROS was historically considered an unwanted by-product of cellular metabolism, however recent work has demonstrated several important roles of ROS as a cell signaling mediator and also roles in oncogenesis, including in AML^38, 39^. FLT3/ITD^+^ AML has increased ROS through several mechanisms including the stabilization expression of the NOX subunit p22phox, increased expression of NOX4, and binding of Rac1-GTP to STAT5^7, 8, 40^. One important role is the inactivation of PTPs through reversible oxidation of cysteines leading to the formation of disulfide bridges and inactivation of the enzyme^41^. As demonstrated here, IB treatment can restore PTP activity through inhibition of ROS leading to decreased tyrosine-phosphorylated STAT5 at nanoMolar doses. This may serve as an attractive STAT5 inhibitor not only for FLT3/ITD^+^ AML but more broadly for other leukemia and myeloproliferative neoplasms with constitutive STAT5 activation to be tested in future studies. Furthermore the combination of ROS inhibition with pimozide conferred a synergistic cell death at sub-therapeutic doses of each drug, demonstrating a potentially useful combination therapy.

Like ROS, Ca^2+^ is also an important mediator of cell signaling. There exists an intimate interconnection of Ca^2+^-containing organelles including endoplasmic reticulum, lysosomes, Golgi, and mitochondria. The ER and mitochondria have multiple contact sites that allow transfer of Ca^2+^. Ca^2+^ release at high doses from the ER to mitochondria may trigger apoptosis through mitochondrial swelling, cytochrome C release, caspase cleavage and mitochondrial rupture. Importantly, lysosomes have recently been identified as an intracellular Ca^2+^ store^42^. Lysosomal Ca^2+^ efflux may also cause a robust release of Ca^2+^ from the ER, likely through the activation of IP_3_ and ryanodine receptors on the ER^43, 44^. This may explain the robust Ca^2+^ release caused by IB, which is similar in intensity to that of the SERCA inhibitor thapsigargin. Peshakov *et al*. confirmed a selective cytotoxic and anti-proliferative effect of Ca^2+^ overload and caspase-induced apoptosis in AML cells using a combination of curcumin and carnosic acid, however the authors did not test AML cells with the FLT3/ITD mutation, so it is unknown if this strategy has greater effects on that subset of AML^45^.

There are important implications regarding the underlying mechanism of IB-induced FLT3/ITD^+^ AML cell death that remain to be determined. It is unclear at this time why IB would preferentially target the lysosome, however we hypothesize that this is due to “lysosomal sequestration”, in which hydrophobic amines such as imipramine accumulate in highly acidic environments (i.e. lysosomes) where they become protonated and are unable to diffuse out of the organelle^46^. This mechanism would not be specific to FLT3-ITD^+^ AML cells. If this is true, then IB must possess a unique property to induce lysosomal Ca^2+^ release as compared to its pro-drug imipramine, which did not induce a cytoplasmic Ca^2+^ release in our experiments. Another important issue is the question of IB’s selectivity for FLT3/ITD^+^ cells. One possibility is that mitochondria are more sensitive to Ca^2+^ overload compared to FLT3/ITD^neg^ AML cells. In addition to STAT5 inhibition, pimozide, a neuroleptic agent, also induces multiple metabolic perturbations including blocking T-type calcium channels which may be responsible for synergy with IB. T-type calcium channels can alter cell cycle dynamics through the action potential generated by a calcium spike. When ER calcium release is accelerated by IB treatment, this hyperpolarization would normally be associated with relieved inactivation of T-type channels. However, pharmacologic inactivation of T-type channels with pimozide could prevent cell proliferation that would normally proceed through calcium mediated p53 inactivation, activation of p21, and release of CDK2 to promote S phase transition. Finally, a safe and efficacious *in vivo* therapeutic approach is needed. Ca^2+^ release as a therapeutic target is currently being tested by using a pro-drug of the SERCA inhibitor thapsigargin (mipsagargin, G-202) in clinical trials for prostate cancer and hepatocellular carcinoma^47^. In this study, we have demonstrated that the imipramine/triphenylmethane conjugate agent imipramine blue possess potent and selective cytotoxic effects alone and in combination with pimozide for FLT3/ITD^+^ AML. The potential clinical utility of this combination remains to be determined. IB acts through a dual mechanism involving STAT5 inhibition and lysosomal Ca^2+^ release at nM concentrations. IB thus represents an exciting new agent and chimeric prototype drug with promise for future development in treating high risk subsets of AML.

## Electronic supplementary material

Supplemental Information
