# Supplementary material for: Imipramine blue sensitively and selectively targets FLT3-ITD positive acute myeloid leukemia cells
Source: Sci Rep. 2017 Jun 30;7:4447. doi: 10.1038/s41598-017-04796-1 (PMC5493614; doi:10.1038/s41598-017-04796-1)
Supplement: Supplementary file 1 — Supplemental Information [file 41598_2017_4796_MOESM1_ESM.docx]

**Imipramine blue sensitively and selectively targets FLT3-ITD positive acute myeloid leukemia cells**

Jonathan Metts^1,2^, Heath L. Bradley^1,2^, Zhengqi Wang^1,2^, Neil P. Shah^3^, Reuben Kapur^4^, Jack L. Arbiser^5,6^, Kevin D. Bunting^1,2^

^1^Department of Pediatrics, Division of Hem/Onc/BMT, Emory University, Atlanta, GA, USA, ^2^Aflac Cancer and Blood Disorders Center, Children’s Healthcare of Atlanta, Atlanta, GA, USA, ^3^Department of Medicine, University of California San Francisco, San Francisco, CA USA, ^4^Department of Pediatrics, Indiana University, Indianapolis, IA USA, ^5^Department of Dermatology, Emory University, Atlanta, GA, USA, ^6^Atlanta Veterans Administration Medical Center

Address correspondence to:

Kevin D. Bunting, Ph.D.

Department of Pediatrics

Division of Hem/Onc/BMT

Aflac Cancer and Blood Disorders Center

Emory University

1760 Haygood Dr. NE, HSRB E308

Atlanta, GA 30322

Tel: 404-778-4039

Email: Kevin.bunting@emory.edu

**Supplementary Figures and Legends:**

**
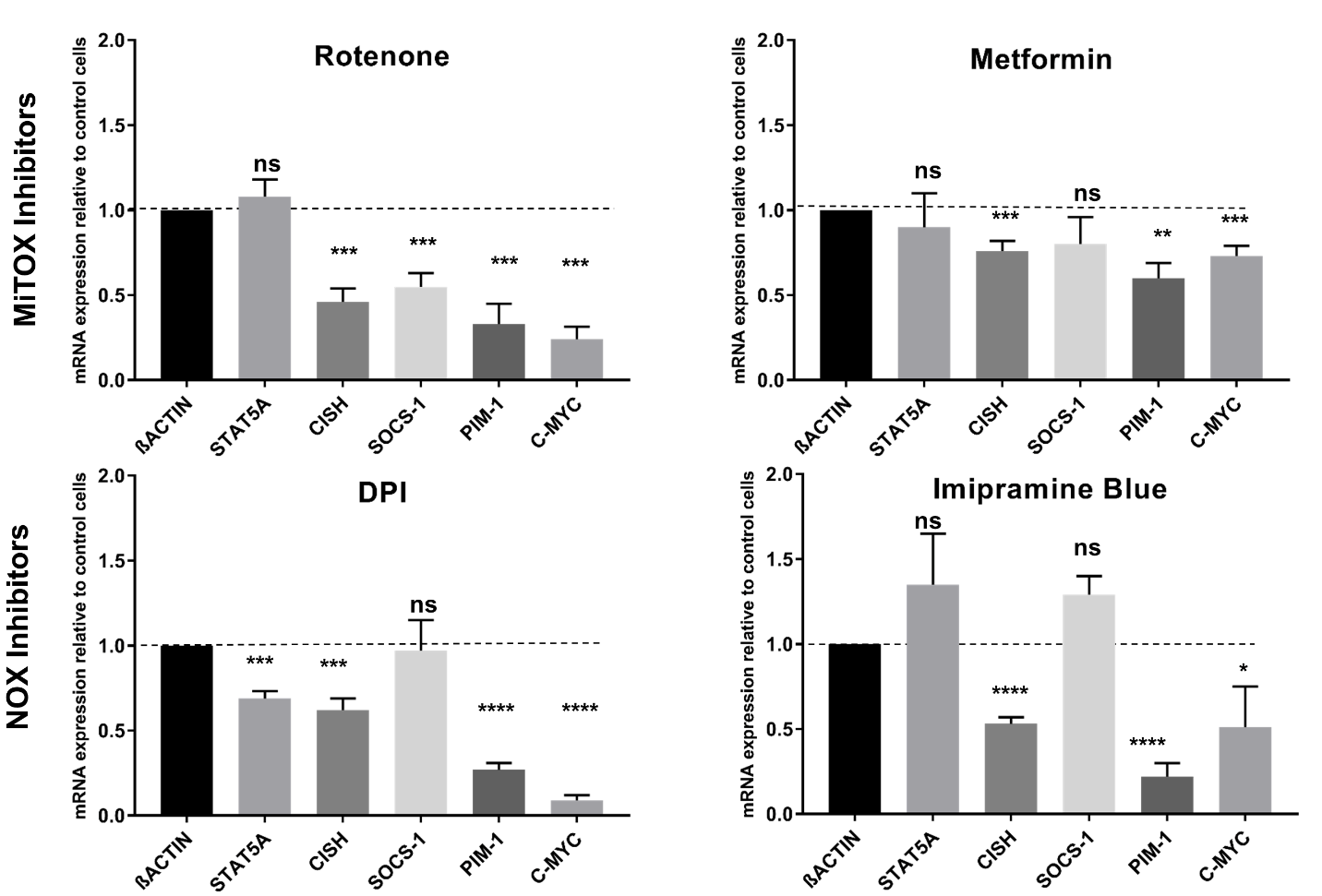
**

**Figure S1. ROS inhibitors decrease expression of STAT5 target genes in MV4-11 cells.** MV4-11 cells were treated with MiTOX inhibitors (rotenone 10 µM, metformin 10 mM) or NOX inhibitors (DPI 10 µM, IB 250 nM) for 4 hours prior to lysis, reverse transcription, and qRT-PCR with β-Actin as the housekeeping control. All agents caused decreased expression of STAT5 target genes. NOX inhibitors drastically reduced expression of downstream genes Pim-1 and Myc, which play important roles in leukemogenesis. (n=3 for each condition). (* p<0.05, ** p<0.01, ns: p>0.05).

**
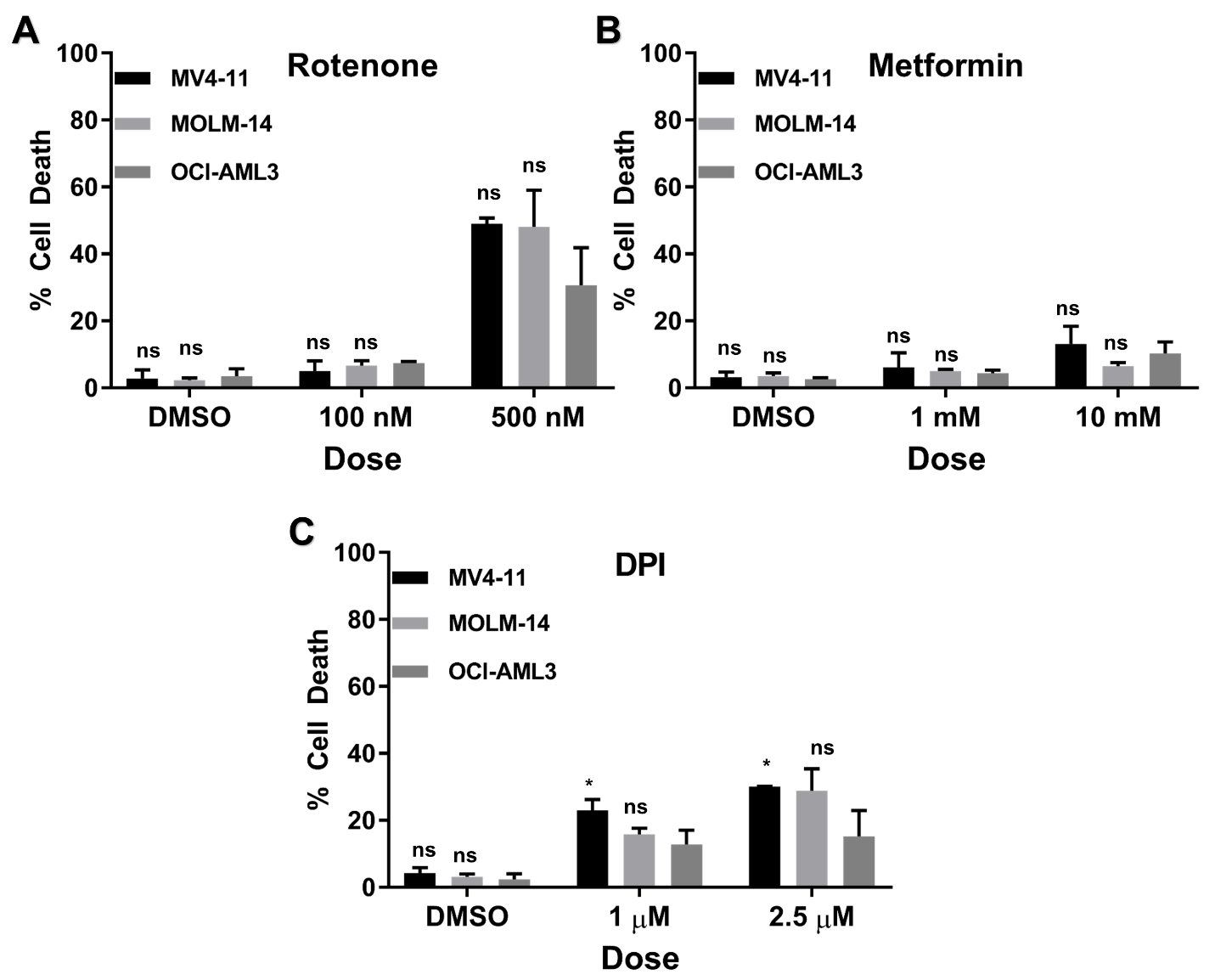
**

**Figure S2. Rotenone, metformin and DPI do not have selective cytotoxicity for FLT3/ITD^+^ AML cell lines.** After 48 hours drug treatment, cytotoxicity was assessed by trypan blue exclusion assay. In contrast to IB, cytotoxicity of other ROS inhibitors to OCI-AML3 (FLT3/ITD^neg^) was not different compared to cytotoxicity of MV4-11 and MOLM-14 cells (FLT3/ITD^+^) (n=3 for each condition) (* p<0.05, ns: p>0.05).
